# Nationwide spatial dynamics of taeniasis in Thailand: declining prevalence but shifting focus and One Health risk factors across 2008–2014

**DOI:** 10.1186/s13071-025-06868-y

**Published:** 2025-07-09

**Authors:** Pornphutthachat Sota, Kefyalew Addis Alene, Thitima Wongsaroj, Sirikachorn Tangkawattana, Archie C. A. Clements, Banchob Sripa

**Affiliations:** 1 School of Animal Technology and Innovation, Institute of Agricultural Technology, Suranaree University of Technology, Nakhon Ratchasima, Thailand; 2 Tropical Disease Research Center, Khon Kaen University, Khon Kaen, Thailand; 3 School of Population Health, Faculty of Health Sciences, Curtin University, Perth, WA Australia; 4 Geospatial and Tuberculosis Team, The Kids Research Institute Australia, Perth, WA Australia; 5 Department of Disease Control, Ministry of Public Health, Nontabuti, Thailand; 6 Department of Veterinary Pathobiology, Faculty of Veterinary Medicine, Khon Kaen University, Khon Kaen, Thailand; 7 School of Biological Sciences, Queen’s University Belfast, Belfast, UK; 8 Department of Tropical Medicine, Faculty of Medicine, Khon Kaen University, Khon Kaen, Thailand

**Keywords:** Taeniasis, Thailand, One Health, Spatial distribution, Geostatistics, Prevalence

## Abstract

**Background:**

The prevalence of taeniasis in Thailand has decreased over the past six decades. However, it remains a public health concern, particularly in focal areas, especially along the border regions where migration between Thailand and neighboring endemic countries is frequent. Spatial distribution analysis provides a useful method for identifying high-risk areas and implementing targeted integrated control measures. This study aimed to examine the spatial patterns of taeniasis in 2008 and 2014, along with their associated One Health risk factors at the sub-district level.

**Methods:**

National surveys of helminthiases and taeniasis were conducted in Thailand in 2008 and 2014. We used data from these surveys and integrated publicly available spatial covariates. A Bayesian spatial model with geostatistical random effects and covariates, implemented using integrated nested Laplace approximation (INLA), was applied to predict the spatial distribution of taeniasis in each survey year.

**Results:**

The prevalence of taeniasis in 2008 was 0.9% (95% CI 0.7–1.1%), while in 2014, it decreased to 0.5% (95% CI 0.4–0.6%). In 2008, higher prevalence was observed in the north, northeast, and parts of the west. In contrast, the 2014 predictions showed a more focal distribution, especially in the western border regions of Thailand, near the Myanmar border. Bangkok was identified as another hotspot, warranting further investigation. One Health factors, including human population density, livestock (cattle and pig) density, antimicrobial use in livestock, and environmental factors (including altitude, precipitation, travel time to cities and healthcare facilities, and normalized difference vegetation index [NDVI]), were associated with high prevalence of taeniasis.

**Conclusions:**

One Health factors significantly influenced the spatial distribution of taeniasis in Thailand. High-risk areas, particularly along the Thai–Myanmar border, require integrated control efforts that involve cross-border collaboration. Cooperation between the Ministry of Public Health and the Department of Livestock Development will be crucial for more effective control measures.

**Graphical Abstract:**

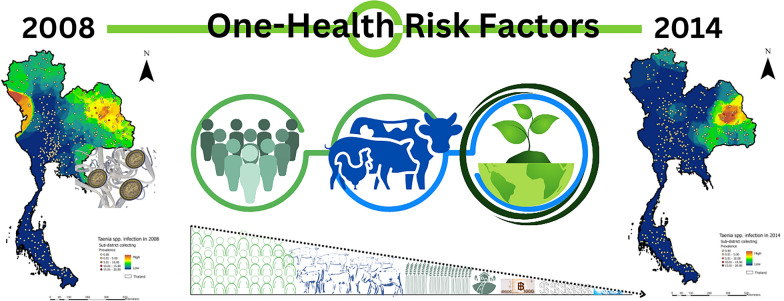

## Background

*Taenia* spp. are zoonotic parasites that include *Taenia solium* (pork tapeworm), *T. saginata* (beef tapeworm), and *T. asiatica* (Asian tapeworm). These parasites cause two major diseases: taeniasis, an intestinal infection, and cysticercosis, a tissue-invasive disease caused by *T. solium* larvae [1]. *Taenia* infections affect the health and economic well-being of millions globally and impose significant economic burdens on the livestock industry [2–6]. Among these species, *T. saginata* is widespread in Thailand and globally, affecting an estimated 60–70 million people [7–9]. Countries neighboring Thailand, including Lao People’s Democratic Republic (Lao PDR) and Myanmar, report a high prevalence of taeniasis, making it a major public health concern in the Southeast Asia region [10–14]. Neurocysticercosis, caused by *T. solium* larvae invading the brain, accounted for the loss of 1.61 million disability-adjusted life years (DALYs) globally in 2017 [15]. In response, the World Health Organization (WHO) aims to eliminate *T. solium* in highly endemic settings by 2030 [16].

Human taeniasis typically results from consuming raw or undercooked meat infected with *T. solium*, *T. saginata*, or *T. asiatica*, while cysticercosis occurs through the ingestion of *T. solium* eggs. Although taeniasis is often asymptomatic, adult worms can develop in the intestinal lumen and, in severe cases, migrate to vital organs such as the brain [17, 18]. As a zoonotic disease, taeniasis poses unique challenges for surveillance and control. Nationwide surveys conducted from 1957 to 1999, and later until 2019, indicate a decreasing prevalence of taeniasis and cysticercosis; however, underreporting remains a significant issue [19]. Despite the decline, after over 70 years of prevention and control efforts, taeniasis continues to pose a health risk to humans and animals [19]. Epidemiological studies and geospatial analyses are crucial for designing targeted national control programs [20]. Understanding the spatial distribution of *Taenia* spp. can facilitate the development of more effective, geographically focused interventions for both human and animal hosts.

This study aimed to investigate the spatial distribution and underlying risk factors associated with *Taenia* spp. infections in Thailand, leveraging nationwide prevalence data from 2008 and 2014. Through the One Health framework, we integrated human behavioral patterns, animal-related factors, and environmental conditions to identify their combined influence on infection dynamics. By uncovering these associations, our findings provide critical insights to inform targeted and sustainable control strategies for mitigating taeniasis transmission in Thailand.

## Methods

### Study setting and data collection

Thailand, located in the central part of Southeast Asia, has a total land area of 513,120 km^2^. It shares borders with Myanmar to the west, Cambodia to the southeast, Lao PDR to the north and east, and Malaysia to the south. The National Helminth Control Program, under the Division of General Communicable Diseases, Department of Disease Control, Ministry of Public Health, conducted nationwide helminthiasis surveys in 2008 and 2014. These surveys covered all 12 regional health offices (RHOs) and 76 provinces of Thailand. A total of 10,301 stool samples were collected in 2008, and 9712 samples were collected in 2014. All participants were residents of Thailand. Fecal samples were examined using the modified Kato–Katz technique. Morphological identification of *Taenia* eggs was performed by experienced technicians and verified by experts at the Division of General Communicable Diseases, Department of Disease Control, Ministry of Public Health, Nonthaburi, Thailand [21–23]. This study analyzed a subset of the national survey data, focusing specifically on *Taenia* spp. infections. As the analysis was conducted on existing survey data, no additional human ethics approval was required.

### Spatial analysis

We included environmental, healthcare access, and zoonotic covariates as independent variables in our statistical model. These covariates were selected based on their availability at high spatial resolution and their plausibility of association with *Taenia* spp. infection risk, as supported by previous studies. The variables included mean annual temperature, precipitation, altitude, population density, accessibility to healthcare facilities and cities, pig density (general and stratified by raising systems: intensive, extensive, and semi-extensive), cattle density, and the quantity of antimicrobial usage in the livestock sector (Table 1).

**Table 1:** Data sources and definitions of covariates

Covariates	Data sources	Definitions
Altitude	Shuttle Radar Topography Mission (SRTM)	Elevation of the earth land surface (km)
Precipitation	WorldClime	Annual mean rainfall (mm)
Travel time to city	Malaria Atlas Project (MAP)	Travel time in minutes to the nearest city with a population of more than 50,000
Population density	WorldPop	Number of people/km^2^ (grid)
Access to healthcare facilities	Malaria Atlas Project (MAP)	Walking travel times in minutes to the nearest health facility
Normalized difference vegetation index (NDVI)	United States Geological Survey (USGS) EROS Data Center	The density of green on a patch of land, the distinct colors (wavelengths) of visible and near-infrared sunlight reflected by the plants are observed
Pig density	Gridded Livestock of the World v2.0 (2008), Harvard Dataverse (2014)	The overall distribution of pig raised head/km^2^
Pigs raised in an intensive system	Gridded Livestock of the World v2.0 (2008)	Largely confined, although intensive outdoor management is widely practiced in some parts of the world. Typically > 100 pigs. Some feed inputs (grains generally) may be home-produced but significant purchases either as straights or ready-mixed
Pigs raised in an extensive system	Gridded Livestock of the World v2.0 (2008)	Pig usually unconfined. Typically < 10 pigs. Scavenging supplemented with household waste
Pigs raised in a semi-intensive system	Gridded Livestock of the World v2.0 (2008)	Usually confined, sometimes with partial scavenging. Typically 10–100 pigs. Home-produced or collected feeds with, in some cases, purchased supplements
Cattle density	Gridded Livestock of the World v2.0 (2008), Harvard Dataverse (2014)	The overall of distribution of cattle raised head/km^2^
Antimicrobial usage in livestock sector	Gridded Livestock of the World v2.0 (2008, 2014)	Antimicrobial consumption distributions for estimates of antimicrobial consumption in cattle, chickens, and pigs

Climate variables, including mean annual temperature and precipitation, were obtained from the WorldClim database (worldclim.org). Altitude data were sourced from the Shuttle Radar Topography Mission (SRTM). Data on travel time to the nearest city and healthcare facility (in minutes) were retrieved from the Malaria Atlas Project [24]. Population density data were obtained from WorldPop (worldpop.org), and normalized difference vegetation index (NDVI) data were downloaded from the United States Geological Survey (USGS) EROS Data Center.

Livestock-related data, including pig and cattle density and antimicrobial usage in 2008, were obtained from the Gridded Livestock of the World v2.0 dataset [25, 26]. Data for pig and cattle density in 2014 were retrieved from the Harvard Dataverse repository [27]. Administrative boundary shapefiles for Thailand were downloaded from the DIVA-GIS website (www.diva-gis.org).

The *Taenia* spp. infection prevalence data were geolocated to the smallest administrative units (sub-districts) using ArcGIS Pro (ESRI, Redlands, CA, USA), a geographical information system (GIS) software. Population-weighted centroids of the sub-districts were used to link the prevalence data with potential covariates. Detailed information, including data sources for the covariates, is provided in Table 1. To assess multicollinearity among candidate variables, variance inflation factors (VIFs) were calculated. The VIF values of all variables that remained were less than the generally accepted cutoff point of 10. Mean annual temperature and altitude both have comparatively high VIF values (9.2 and 8.1, respectively); only one of these variables was used in the final model because of their strong correlation. Based on biological plausibility and an association with the probability of infection with *Taenia* spp., altitude was included in our final candidate variables. Therefore, to prevent redundancy, mean annual temperature was not included in the multivariable model.

Spatially continuous predicted prevalence estimates for *Taenia* spp. across Thailand were generated for the years 2008 and 2014 using Bayesian model-based geostatistics (MBG). A spatial binomial regression model was developed, with *Taenia* spp. prevalence as the outcome variable. All candidate covariates were included simultaneously in the model to estimate their adjusted associations with infection prevalence. Statistical significance was assessed based on 95% Bayesian credible intervals (CrI), with variables considered significant if their CrI did not include zero. This multivariable modeling approach was selected to account for both spatial structure and the combined effects of multiple predictors. The selected covariates included precipitation, altitude, population density, accessibility to healthcare facilities and cities, pig density (including intensive, extensive, and semi-extensive raising systems), cattle density, and antimicrobial usage in the livestock sector.

The number of individuals infected with *Taenia* spp. at each surveyed location *j* was assumed to follow a binomial distribution:

$$Y_{j} \sim {\text{Binomial }}\left( {n_{j} ,p_{j} } \right);$$

where *n* is the total number of participants, and *p* is the predicted prevalence of *Taenia* spp. infection at location *j*. Prevalence at each location was related to a linear predictor via a logit link function, whereby

$$logit(p_{j} ) = a + \sum\nolimits_{Z = 1}^{Z} {\beta_{Z} X_{Z,j} } \zeta_{j}$$

where α denotes the intercept, *β* is the matrix of covariate coefficients, *X* is a design matrix of *z* covariates, and ζj are spatial random effects modeled using a zero-mean Gaussian Markov random field with a Matérn covariance function [28]. The Matérn covariance function is characterized by two parameters: the spatial scale (ρρ), which defines the distance beyond which correlation becomes negligible, and the marginal standard deviation (σσ) [29, 30]. Default priors were applied to the parameters of the spatial random fields [31]. Parameter estimation was conducted using integrated nested Laplace approximation (INLA) [32] and implemented in the R statistical software [33]. Sufficient iterations were performed for each variable of interest to ensure a thorough characterization of the posterior distributions.

The model was applied to predict *Taenia* spp. prevalence in unsampled locations across Thailand using a 1 km^2^ resolution prediction grid. At each prediction location, the intercept, the spatial random effect values, and the products of the covariate coefficients with the observed values of the spatially varying fixed effects were summed and then back-transformed from the logit scale to the prevalence scale. The results were imported into GIS software to create predicted prevalence maps.

For model validation, the conditional predictive ordinates (CPO) and probability integral transform (PIT) statistics were calculated [34]. The same multivariable Bayesian spatial binomial regression model was applied separately to *Taenia* spp. infection data for 2008 and 2014 using an MBG framework. All candidate covariates were included simultaneously in the model to estimate adjusted associations. Statistical significance was assessed using 95% Bayesian CrI, where variables were considered significant if the CrI did not include zero. For both years, model interpretation was based on a combination of variable significance and model fit using the Watanabe–Akaike information criterion (WAIC). In instances where no variables reached statistical significance, final model selection prioritized WAIC and biological plausibility to ensure epidemiological relevance. The best-fitting model was identified as the one with the lowest WAIC score [35].

## Results

### Prevalence

**Fig. 1:**
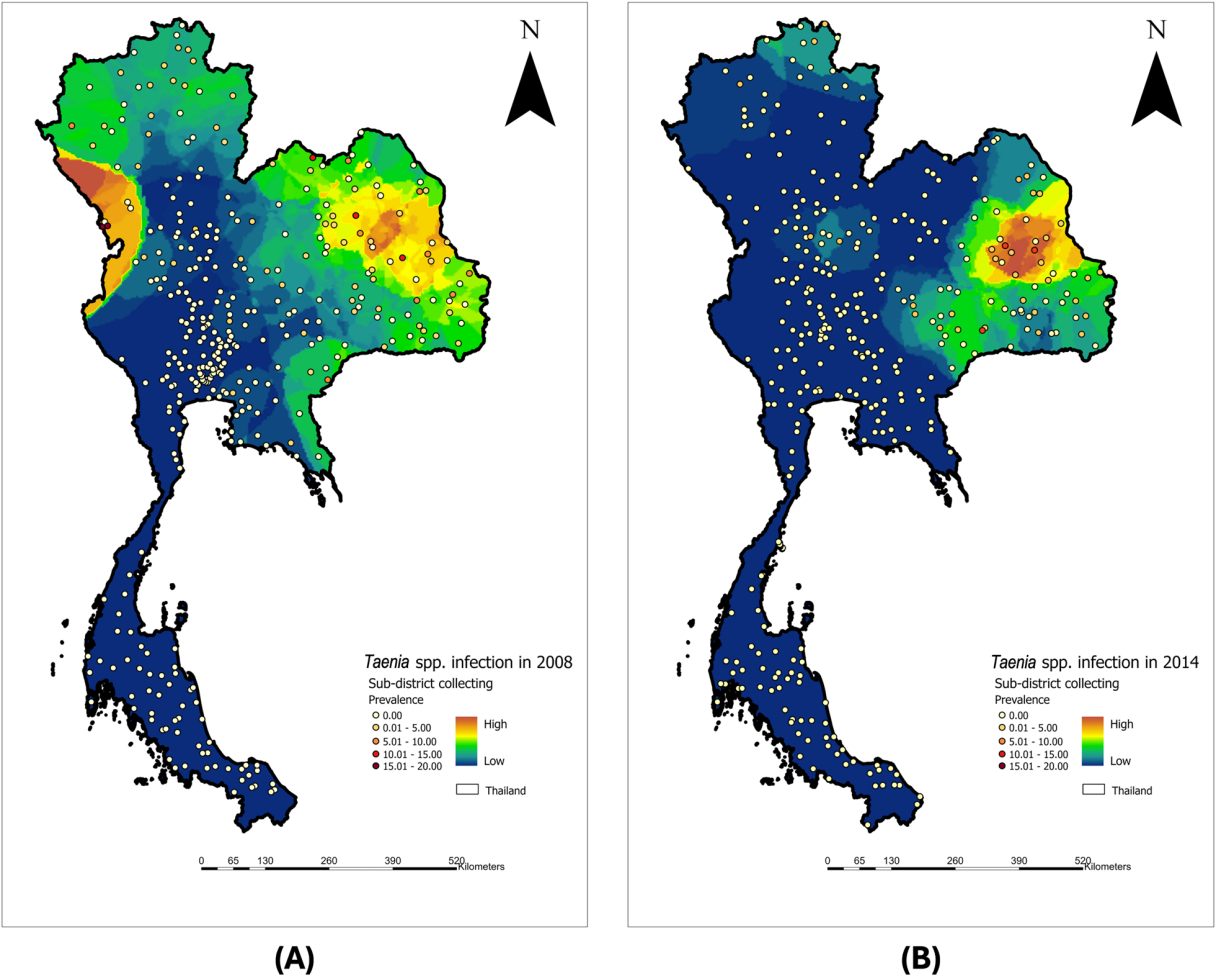
Sample collection points at the sub-district level. *Taenia* spp. infection rates are 0.9% in 2008 (**A**) and 0.5% in 2014 (**B**). In 2008, regions in the northeast, northwest, and eastern parts of Thailand had high infection rates. Alternatively, 2014 had a particularly high infection rate in northeastern Thailand

The national prevalence of *Taenia* spp. infection in Thailand was 0.9% (95% CI 0.7–1.1) in 2008, declining to 0.5% (95% CI 0.4–0.6) in 2014. Among 356 sub-districts surveyed in 2008, *Taenia* spp. infections were detected in 68 sub-districts. Infected participants were identified across all regions except the southern region. The highest prevalence in 2008 was observed in Mae Tao sub-district (20.0%) and Phra That Padang sub-district (18.0%), both located in Mae Sot District, Tak Province, along the Thai–Myanmar border (Fig. 1A).

In the 2014 survey, *Taenia* spp. infections were detected in 35 out of 329 surveyed sub-districts. Nong Khun Yai Sub-district in Nong Phok District, Roi et Province, had the highest prevalence of *Taenia* spp. infection at 9.5%, followed by Lak Mueang Sub-district in Kamalasai District, Kalasin Province, with a prevalence of 7.1% (Fig. 1B).

### Predicted spatial distribution of *Taenia* spp. infection

**Fig. 2:**
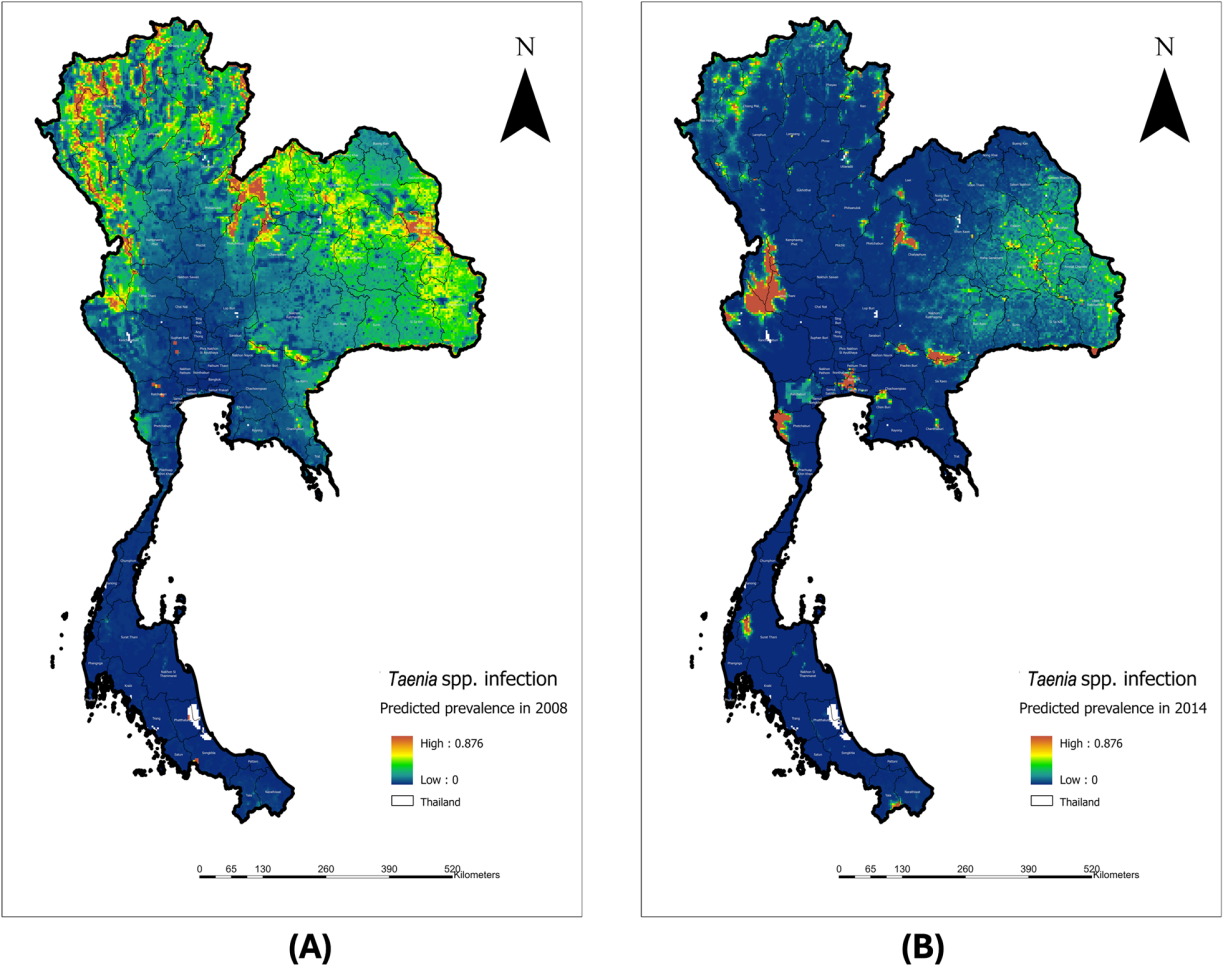
Predicted mean probability of *Taenia* spp. infection in humans in Thailand in 2008 (**A**) and 2014 (**B**)

In 2008, areas with a higher predicted prevalence of *Taenia* spp. infection were primarily located in the north, northeast, west, and parts of East Thailand. The most affected regions were broadly distributed in the north, northeast, and parts of the west near the Myanmar border (Fig. 2A). Predicted prevalence was very low in the southern region.

**Fig. 3:**
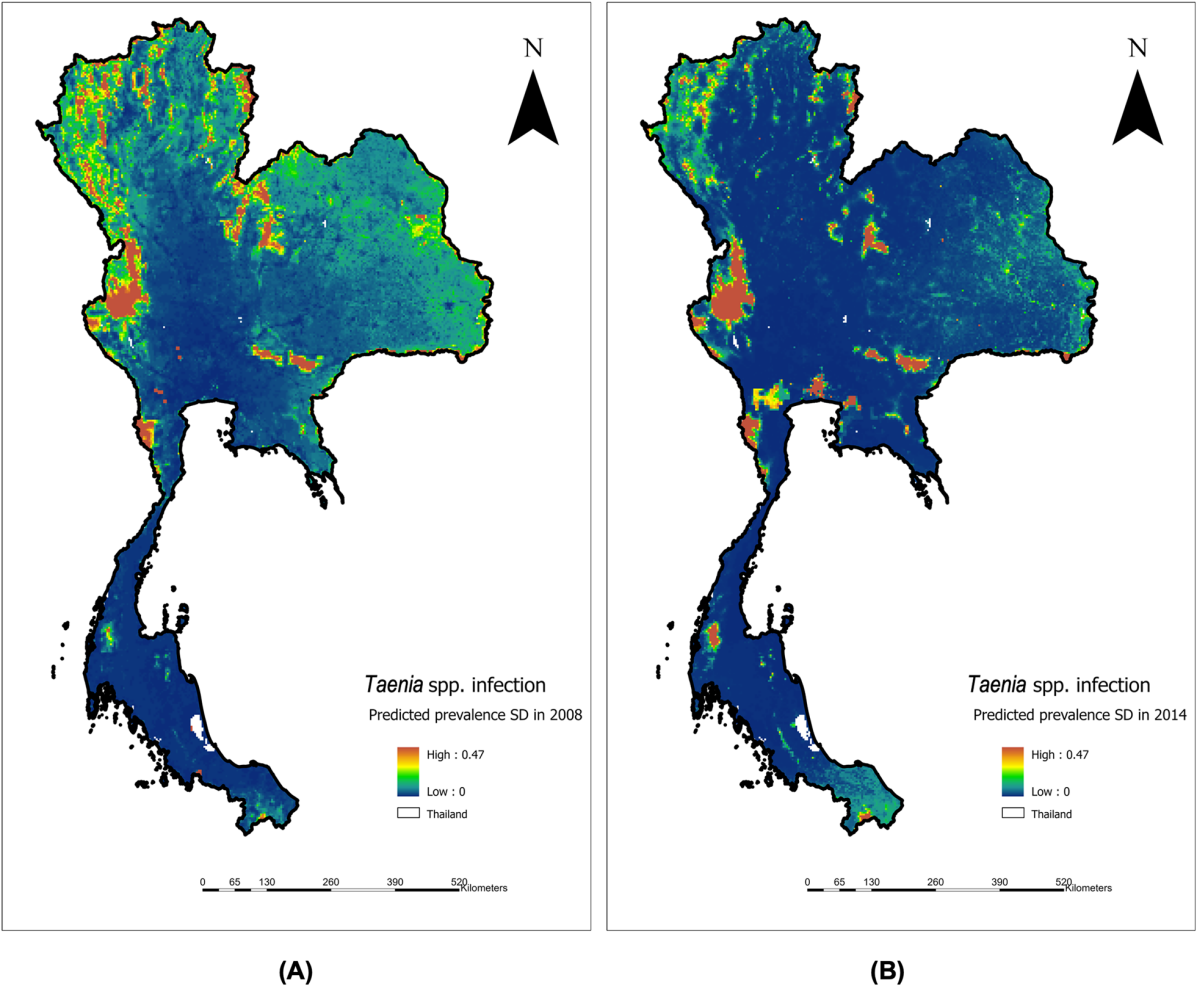
SD probability of *Taenia*. spp infection in 2008 (**A**) and 2014 (**B**)

By 2014, the prevalence of *Taenia* spp. infection had decreased, and the predicted areas of high prevalence were more localized (Fig. 2B). Hotspot areas were identified in Tak and Kanchanaburi provinces, both bordering Myanmar. Notably, Bangkok, Thailand’s capital city, emerged as a predicted hotspot. In the northeastern region, only Nakhon Ratchasima and Chaiyaphum provinces were identified as hotspots. As in 2008, the southern region remained a low-prevalence area for *Taenia* spp. infection. The SD probability of *Taenia*. spp. infection in 2008 and 2014 are shown in Fig. 3A and 3B, respectively.

### Predictors of *Taenia* spp. infection in 2008 and 2014

The final multivariable Bayesian spatial models for *Taenia* spp. infection in 2008 and 2014 are presented in Table 2 and Table 3, respectively. In 2008, significant predictors of *Taenia* spp. infection included population density (95% CI –2.6, –0.8), cattle density (95% CrI 0.1, 2.2), and the quantity of antimicrobial usage in the livestock sector (95% CrI –2.0, –0.2). These variables were statistically significant, based on 95% Bayesian credible intervals that did not include zero, and represent adjusted associations from the multivariable model.

**Table 2:** Regression coefficient mean and 95% credible intervals (CrI) of covariates included in a Bayesian spatial model with binomial response for the prevalence of *Taenia* spp. infection in 2008 and 2014

Covariates	2008 Regression coefficient mean 95% (CrI)	2014 Regression coefficients mean 95% (CrI)
*z* intercept	−5.0 (−33.2 to 27.8)	−7.3 (−38.5 to 30.3)
Altitude (m)	0.6 (0.0 to1.2)	0.0 (−1.6 to 1.2)
Precipitation (mm)	−0.2 (−0.7 to 0.3)	−0.3 (−1.5 to 0.9)
Travel time in minutes to city	−0.5(−1.2 to 0.1)	0.0 (−1.2 to 1.3)
Population density (i.e., number of people/km^2^)	−1.6 (−2.8 to −0.6)*	0.2 (−0.6 to 0.8)
Travel time in minutes to healthcare facility	0.0 (−0.8 to 0.8)	1.2 (−0.3 to 2.6)
Normalized difference vegetation index	0.0 (−0.4 to 0.4)	−0.7 (−1.2 to −0.2)
Pig density (heads/km^2^)	−0.3 (−0.9 to 0.1)	0.1 (−1.0 to 0.9)
Pigs raised in an intensive system (heads/km^2^)	0.1 (−43.8 to 43.9)	NA
Pigs raised in an extensive system (heads/km^2^)	−0.1(−0.4 to 0.2)	NA
Pigs raised in a semi-intensive system (heads/km^2^)	0.1 (−43.8 to 43.9)	NA
Cattle density (heads/km^2^)	1.1 (0.1 to 2.2)*	−0.4 (−0.9 to 0.1)
Antimicrobial usage in livestock sector (mg/10 km^2^ pixels)	−1.0 (−2.0 to −0.2)*	0.1 (−0.5 to 0.8)

*A variable was considered statistically significant if its 95% credible interval (CrI) did not include zero

**Table 3:** Comparison of models with different covariate combinations, for spatial prediction of *Taenia* spp. Infection in Thailand, 2008 and 2014

Model	2008 WAIC^b^	2014 WAIC^b^
Altitude	366.2	630.9
Altitude + Prec	368.0	556.3
Altitude + Prec + Traveltime	370.0	640.6
Altitude + Prec + Traveltime + Pop	360.6	597.2
Altitude + Prec + Traveltime + Pop + Dist_HF	362.7	752.2
Altitude + Prec + Traveltime + Pop + Dist_HF + NDVI	367.2	236.3
Altitude + Prec + Traveltime + Pop + Dist_HF + NDVI + Pigden	365.4	277.7
Alt + Prec + Traveltime + Pop + Dist_HF + NDVI + Pigden + Cattleden	**NA**	**271.7**
Alt + Prec + Traveltime + Pop + Dist_HF + NDVI + Pigden + Cattleden + ABO	NA	318.8
Altitude + Prec + Traveltime + Pop + Dist_HF + NDVI + Pigden + Piginten	366.1	NA
Altitude + Prec + Traveltime + Pop + Dist_HF + NDVI + Pigden + Piginten + Pigexten	368.7	NA
Altitude + Prec + Traveltime + Pop + Dist_HF + NDVI + Pigden + Piginten + Pigexten + Pigsemi	368.7	NA
Altitude + Prec + Traveltime + Pop + Dist_HF + NDVI + Pigden + Piginten + Pigexten + Pigsemi + Cattleden	369.8	NA
Altitude + Prec + Traveltime + Pop + Dist_HF + NDVI + Pigden + Piginten + Pigexten + Pigsemi + Cattleden + ABO	365.7	NA

*Alt* altitude, *Prec* precipitation, *Traveltime* travel time to city, *Pop* population density, *Dist_HF* distance to healthcare facility, *NDVI* normalized difference vegetation index, *Pigden* pig density, *Piginten* pigs raised in an intensive system, *Pinexten* pigs raised in an extensive system, *Pigsemi* pigs raised in a semi-intensive system, *Cattleden* cattle density, *ABO* antimicrobial usage in livestock sector.

*WAIC* ^b^Watanabe–Akaike applicable information criterion. Bold indicates the selected model.

**Fig. 4:**
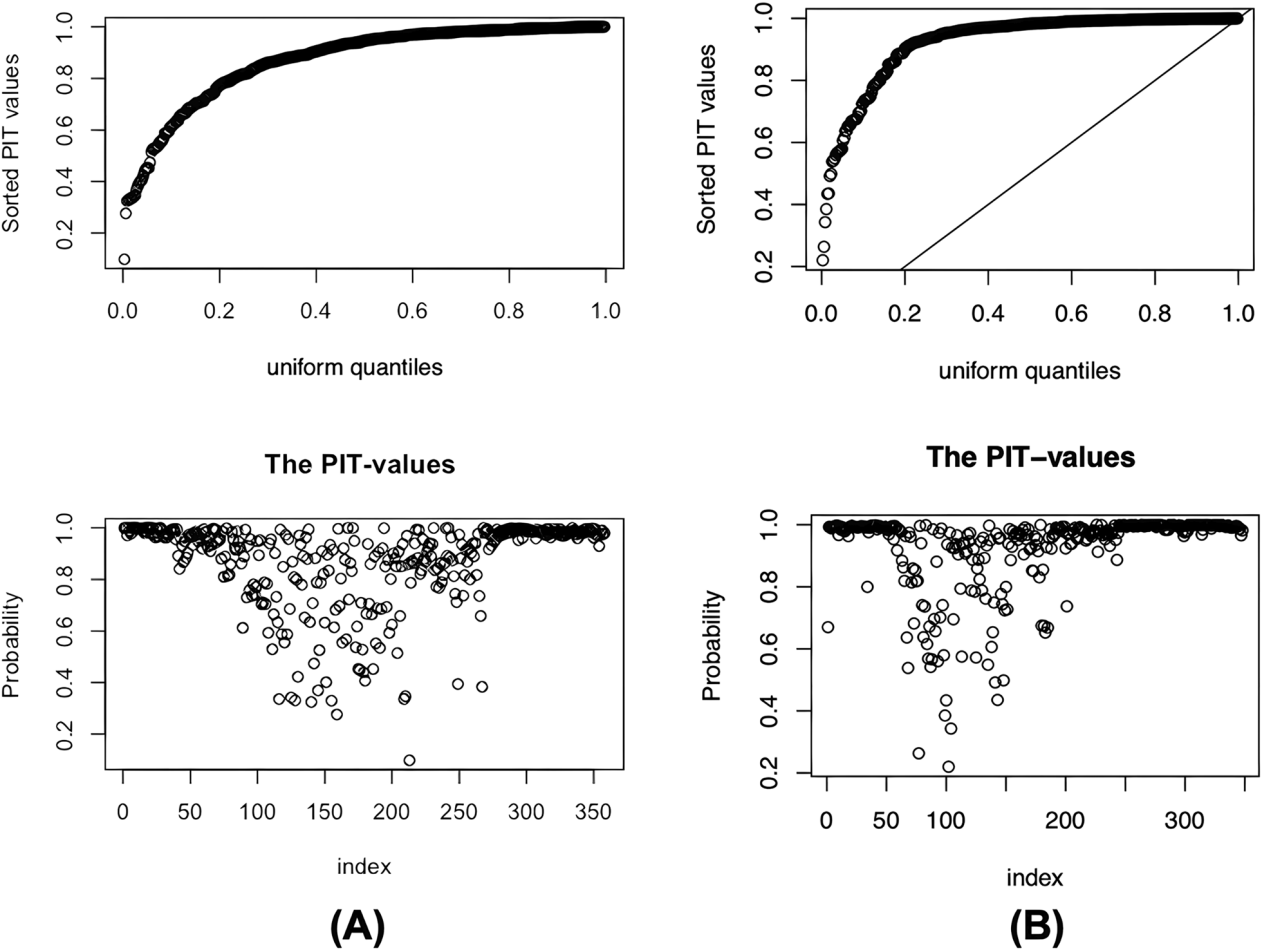
Model validation diagrams of *Taenia* spp. infection in 2008 (**A**) and 2014 (**B**)

In contrast, no covariates in the 2014 model were statistically significant based on CrI. Nevertheless, the final model was chosen based on the lowest WAIC among those that retained biologically meaningful variables, especially those related to livestock density. However, the final model was selected based on the lowest WAIC value among the models that included biologically relevant predictors, particularly livestock density. Notably, while the model with the absolute lowest WAIC (236.3) had slightly better statistical fit, it excluded key epidemiological variables such as pig and cattle density, which are essential for interpreting *Taenia* spp. transmission. In 2008, cattle density was identified as a meaningful risk factor; therefore, to maintain interpretability and consistency across both years, we prioritized the 2014 model that included livestock population density—especially given the well-established role of pigs in *Taenia* transmission. The selected model (WAIC = 271.7) provided a stronger basis for public health interpretation despite the slightly higher WAIC (ΔWAIC = 35) (Table 3). Model validation results, shown in Fig. 4A and B, indicate that the predictive models for both 2008 and 2014, respectively, were well fitted, as evidenced by the probability integral transform (PIT) statistics.

## Discussion

The most common *Taenia* infections in humans are caused by *T. saginata* and *T. solium*, which utilize cattle and pigs as their intermediate hosts, respectively [36, 37]. These zoonotic infections pose dual challenges: a substantial public health burden due to risks of taeniasis and cysticercosis and significant economic losses to livestock industries [7]. This study represents the first comprehensive investigation of the spatial distribution of *Taenia* spp. at a national scale in Thailand. Our findings highlight the complex interplay of human behavioral, social, animal-related, and environmental factors that drive *Taenia* spp. infection. These factors underscore the necessity of a One Health approach, integrating human, animal, and environmental health perspectives to effectively tackle this issue.

Past national surveys have shown a steady decline in *Taenia* spp. infection prevalence in Thailand, from 2.5% in 1957 to 0.6% in 2001 [8]. This downward trend is further supported by the findings of our study, with nationwide prevalence estimates of 0.9% in 2008 and 0.5% in 2014. Spatial predictions from 2008 revealed high-prevalence areas concentrated in the north, northeast, and parts of northwestern Thailand near the Thai–Myanmar border, consistent with prior studies [8]. By 2014, these high-prevalence areas had contracted into smaller, more focal hotspots, primarily in Tak and Kanchanaburi provinces near the Thai–Myanmar border, regions previously reported as endemic for *Taenia* spp. [8, 38, 39]. A smaller hotspot was also predicted in parts of the northeast bordering Laos.

In these regions, cultural and behavioral risk factors, such as the consumption of raw or undercooked pork dishes like *lahb*, *loo*, or *koi*, may contribute to the persistence of *Taenia* spp. infection [38]. Kusolsuk et al. [38] documented a prevalence of 2.8% for taeniasis in Tak Province, with most infections attributed to *T. solium*. Additionally, consumption of fresh vegetables potentially contaminated with *Taenia* spp. eggs may act as a secondary transmission pathway [38, 40]. In contrast, the absence of hotspots in Southern Thailand aligns with distinct cultural practices that discourage the consumption of raw or undercooked meat. However, the predicted hotspot in Bangkok warrants further investigation, as no empirical data were collected from this region during the surveys. This anomaly could reflect biases in spatial modeling or unmeasured risk factors, such as urban migration or increased pork consumption patterns.

Population density was identified as a risk factor for *Taenia* spp. infection, consistent with findings from other studies that have demonstrated both positive and negative associations with population density [41]. The observed decline in population density in certain regions may reflect poverty and environmental stress, which drive migration from rural areas to larger cities [42]. Under poverty conditions, households in rural areas often raise and slaughter pigs in backyards without proper meat inspection. Pork serves as an essential protein source for impoverished families, providing sustenance despite limited financial resources [43]. The regions with higher poverty levels, such as parts of the North, Northeast, and West, correspond to areas identified as having a higher prevalence of *Taenia* spp. infection in our study. For instance, Tak and Kanchanaburi provinces, characterized by some of the lowest population densities in Thailand, showed the highest prevalence of taeniasis in 2008 [44, 45]. Additionally, the migration of workers from endemic regions may contribute to increased transmission risks [43].

Travel time to urban centers and healthcare facilities emerged as significant risk factors, highlighting the socioeconomic vulnerabilities of populations in remote areas. Marginalized groups residing far from cities often have limited access to health education, diagnostic services, and treatment, exacerbating the risk of *Taenia* spp. infections [46]. These findings underscore the critical role of improved healthcare access and socioeconomic development in reducing the burden of *Taenia* spp. infections in rural and impoverished regions.

The use of antimicrobial drugs in food-producing animals is essential for treating infectious diseases, ensuring animal welfare, and maintaining productivity [47]. Antimicrobial and antiparasitic drug usage often show parallels, as their sales data strongly correlate with trends in meat production [48]. Additionally, the market shares of these drug types are comparable in volume [49]. For instance, regions with low antimicrobial drug usage are also likely to have low antiparasitic drug usage. The observed negative association between antimicrobial usage and *Taenia* spp. infection prevalence may suggest that areas with limited availability or use of veterinary pharmaceuticals face higher risks of transmission from animals to humans. This is particularly relevant when infections in intermediate hosts are not adequately managed through the use of antiparasitic drugs.

Furthermore, higher cattle density was positively associated with *Taenia* spp. infection risk. This association could be due to the increased probability of transmission from intermediate hosts (cattle) to humans in regions where cattle density is high. Areas with greater cattle populations often exhibit higher beef consumption, further elevating the risk of human infections.

Our study supports previous reports that have identified significant correlations between environmental factors and *Taenia* spp. prevalence. The physical environment plays a crucial role in egg survival, which, in turn, impacts the transmission potential of the parasite [50]. In a review by Wardrop et al. [51], precipitation was identified as a risk factor for *Taenia* spp. infection, a finding that aligns with the results from our models [52].

In the northeastern and northern regions, where several hotspots of *Taenia* spp. infection were identified, *Opisthorchis viverrini* (liver fluke) infection is also endemic. A key component of *O. viverrini* control is mass drug administration with praziquantel [53–57]. Praziquantel or niclosamide are also the drugs of choice for *Taenia* spp. treatment, suggesting that *Taenia* control could be integrated with *O. viverrini* control programs [15, 58].

Half of the population in Tak and Kanchanaburi provinces are immigrants from Myanmar [8]. Studying infections in immigrant populations can provide insights into the disease profiles of their countries of origin. While Myanmar has seen limited studies, McCleery et al. [14] reported a 3.3% prevalence of taeniasis in refugees from Myanmar, indicating that eastern Myanmar may have endemic *Taenia* infections. Refugees could be a significant source of infections in neighboring parts of Thailand [6, 38]. Poor sanitation in refugee communities exacerbates the problem, with Kusolsuk et al. [38] reporting that 14.6% of villagers in Tak defecate in pit latrines or the bush, facilitating the transmission of taeniasis between humans and animals.

Surprisingly, Bangkok and its surrounding areas emerged as a hotspot for *Taenia* spp. infection in 2014. Despite being one of the most densely populated regions in Thailand, Bangkok has limited recent data on *Taenia* infections. Notably, 25.5% of neurocysticercosis patients in Thailand were from Bangkok [59]. The increasing population density in Bangkok is largely driven by two groups: first, individuals from other provinces who migrate to the city for work, and second, the migrant workforce that comes specifically to seek employment in Bangkok [60]. Both groups may contribute to the spread of diseases within the city. Furthermore, the unmet needs of Bangkok's residents, caused by long waiting times and limited transportation, may foster an environment conducive to the undetected spreading of diseases [61]. Our model predictions underscore the need for further data collection and risk assessments in the Bangkok region.

Limitations of this study are that the diagnostic approach used did not allow for the identification of *Taenia* species. Molecular analyses or serological assays capable of detecting the exact *Taenia* species were not conducted, meaning we could not differentiate between infections related to pigs or cattle. Additionally, the lack of interview data may have impacted the validity of the prediction maps, as behavioral factors are important drivers of infection. For future studies, parallel sample collection from both humans and animals in the same regions, along with the inclusion of additional covariate information gathered through questionnaires, will provide a more comprehensive understanding of the drivers of taeniasis in Thailand. Additional limitations include the use of a full-model method without preceding univariable screening, which may have added variables with limited contribution. In 2014, no covariates attained statistical significance; thus, model selection was based on WAIC and theoretical relevance, which may involve interpretive subjectivity. Despite these limitations, the study provides important insights into the spatial and ecological risk factors associated with *Taenia* spp. infection in Thailand and demonstrates the utility of Bayesian model-based geostatistics for informing targeted control strategies.

While the prevalence of *Taenia* spp. infections in Thailand continues to decline, hotspots of infection persist, particularly in remote areas near the Thailand–Myanmar border [38]. Pigs in these areas have been reported to test positive for *T. solium* infection [62, 63], highlighting the potential public health risk associated with this infection in Thailand. Effective meat inspection and hygiene practices are essential to reducing the risk of infection and controlling the transmission of *Taenia* spp. from animals to humans. Given the interconnection between human, animal, and environmental health, a One Health approach is critical for the effective control of taeniasis-cysticercosis. Collaborative efforts between human health authorities and the Department of Livestock Development are necessary to implement such an integrated One Health strategy. Furthermore, there was a deficiency of knowledge regarding taeniasis and cysticercosis [64]. Consequently, it is advisable to implement public education interventions aimed at enhancing knowledge among residents with low socioeconomic status, particularly in the identified hot spot zone.

## Conclusions

Our study examined the characteristics of taeniasis in Thailand at two time points, 2008 and 2014, emphasizing the key role of the One Health approach in identifying risk factors and guiding efforts for the prevention and control of taeniasis. The findings highlight priority areas for targeted interventions to tackle the transmission of taeniasis in Thailand. Additionally, the surveillance and control systems should foster greater collaboration between Thailand and neighboring endemic countries, particularly those with a significant immigrant workforce, to better address cross-border transmission.

## Data Availability

Data supporting the main conclusions of this study are included in the manuscript.
